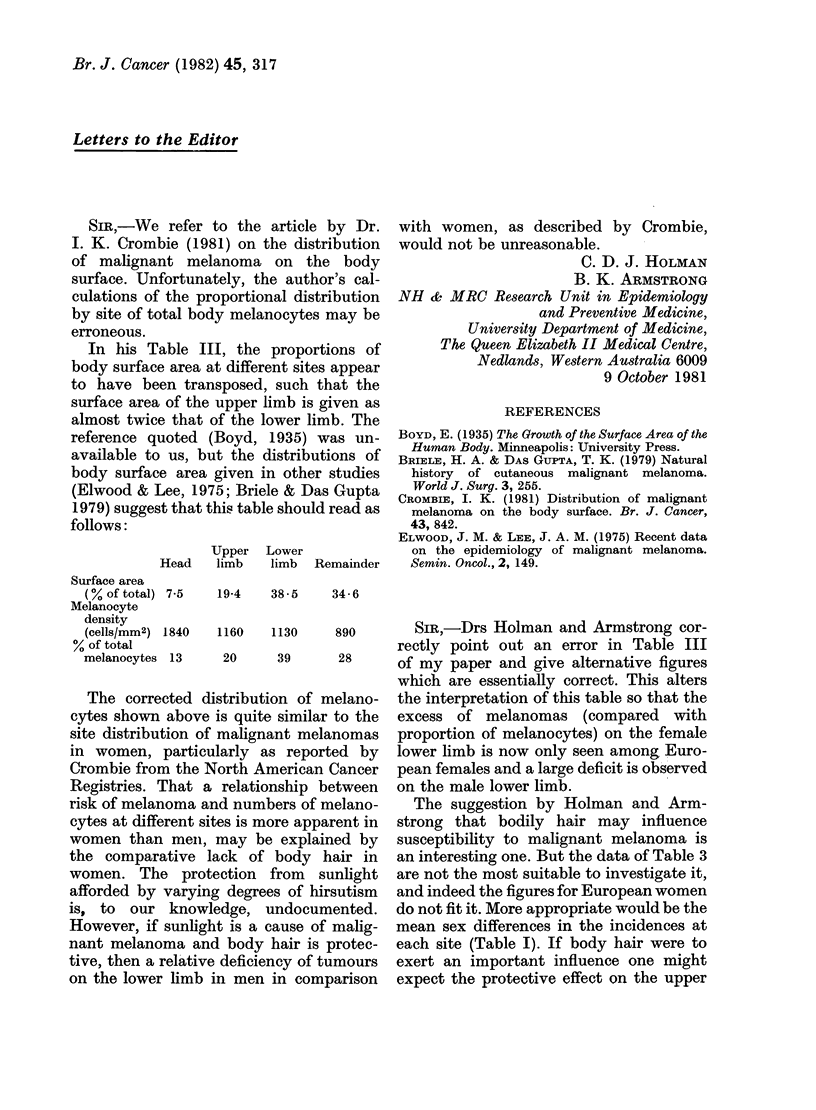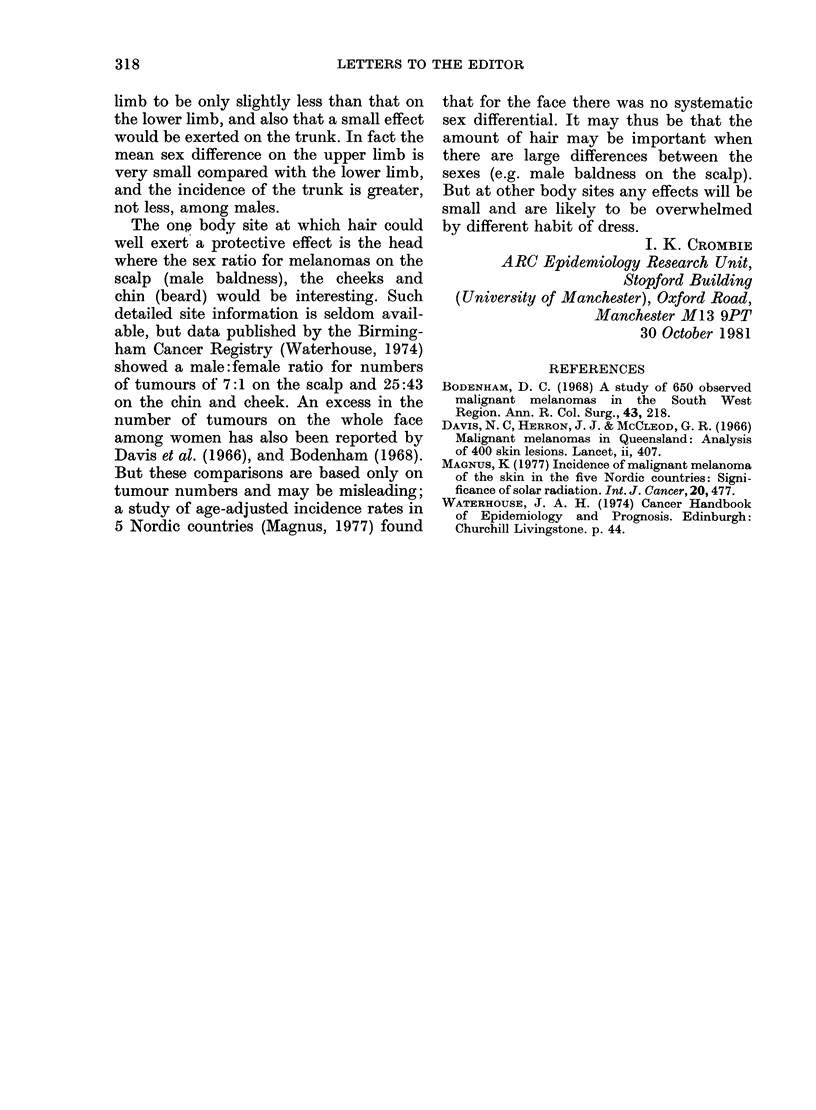# Letters to the Editor

**Published:** 1982-02

**Authors:** I. K. Crombie


SIR,—Drs Holman and Armstrong correctly point out an error in Table III of my paper and give alternative figures which are essentially correct. This alters the interpretation of this table so that the excess of melanomas (compared with proportion of melanocytes) on the female lower limb is now only seen among European females and a large deficit is observed on the male lower limb.

The suggestion by Holman and Armstrong that bodily hair may influence susceptibility to malignant melanoma is an interesting one. But the data of Table 3 are not the most suitable to investigate it, and indeed the figures for European women do not fit it. More appropriate would be the mean sex differences in the incidences at each site (Table I). If body hair were to exert an important influence one might expect the protective effect on the upper limb to be only slightly less than that on the lower limb, and also that a small effect would be exerted on the trunk. In fact the mean sex difference on the upper limb is very small compared with the lower limb, and the incidence of the trunk is greater, not less, among males.

The one body site at which hair could well exert a protective effect is the head where the sex ratio for melanomas on the scalp (male baldness), the cheeks and chin (beard) would be interesting. Such detailed site information is seldom available, but data published by the Birmingham Cancer Registry (Waterhouse, 1974) showed a male:female ratio for numbers of tumours of 7:1 on the scalp and 25:43 on the chin and cheek. An excess in the number of tumours on the whole face among women has also been reported by Davis *et al.* (1966), and Bodenham (1968). But these comparisons are based only on tumour numbers and may be misleading; a study of age-adjusted incidence rates in 5 Nordic countries (Magnus, 1977) found that for the face there was no systematic sex differential. It may thus be that the amount of hair may be important when there are large differences between the sexes (e.g. male baldness on the scalp). But at other body sites any effects will be small and are likely to be overwhelmed by different habit of dress.

I. K. Crombie
*ARC Epidemiology Research Unit,*
*Stopford Building*
(*University of Manchester*), *Oxford Road,*
*Manchester M*13 9*PT*
30 *October* 1981

REFERENCES

Bodenham, D. C. (1968) A study of 650 observed malignant melanomas in the South West Region. Ann. R. Col. Surg., **43**, 218.

Davis, N. C, Herron, J. J. & McCleod, G. R. (1966) Malignant melanomas in Queensland: Analysis of 400 skin lesions. Lancet, ii, 407.

Magnus, K (1977) Incidence of malignant melanoma of the skin in the five Nordic countries: Significance of solar radiation. *Int. J. Cancer*, **20**, 477.

Waterhouse, J. A. H. (1974) Cancer Handbook of Epidemiology and Prognosis. Edinburgh: Churchill Livingstone. p. 44.